# Supposed Case of Passive Congestion of the Brain, with Effusion

**Published:** 1857-07

**Authors:** A. A. Davis

**Affiliations:** Culpeper County, Virginia


### ARTICLE III.

*Supposed Case of Passive Congestion of the Brain, with Effusion.*
By A. A. DAVIS, M. D., of Culpeper County, Va.

On the morning of the 7th of April, I was called to Henry Banks, æt 50, a blacksmith, who had been in bad health for a number of years; found him suffering with severe pain in the epigastric region, sick stomach, &c., caused, as I thought, by a hearty breakfast of fish, dried meats, coffee, &c. On pressure over the stomach, vomiting was induced, carried only to the emptying of the stomach of its contents; but, on the pain still continuing after vomiting, and his bowels being constipated, with slight twitching of the muscles, I gave him

℞—Col. ext. comp., vi grs.
Calomel, v. grs.
Morphine, one-sixth gr.

In a few minutes after swallowing it, the patient was generally convulsed, which soon subsided, but leaving him with stupor, almost to profound coma.

Counter irritation was made by mustard plasters to the whole spine and extremities; the feet placed in warm mustard water; stimulating clysters to be used repeatedly; the coma existing during the day; the pulse becoming feeble and irregular; the skin cool and moist; breathing slow and easy.

7th. 5 P. M.—Patient still comatose; circulation feeble and depressed; pulse 80, soft and feeble; skin cool and moist; bowels had acted well; urine high colored: ordered vesication of the extremities, by means of blistering cerate and hot turpentine; the bowels to be kept open with extract colocynth and calomel.

8th. 7 A. M.—Patient no better; still comatose and depressed; vesicants had acted well; bowels open; in which condition he remained until the

9th. 7 A. M.—Patient shows symptoms of reactions; pulse 80, with more body; skin warm and moist; bowels open; mind wandering, and some restlessness; urine still red: ordered counter irritant to be kept up; but fearing too great reaction, ordered cold applications to be kept to the head.

5 P. M.—Reaction strong; pulse 100, full and strong; skin dry and hot; tongue red and dry; delirious, with desire to get up; bowels opened by injections.

℞—Ext. col. comp. xxx. grs.
Calomel xx. grs.
Ipecac x. grs.

M. Pulv. x.; give one *ter die.*

℞—Veratrum Viride, v. M. every three hours.

10. 7 A. M.—Patient more rational; pulse 80, soft and full; skin warm and moist; tongue less red, but furred; bowels open; recognizes individuals.

Treatment the same, using vi. M. Veratrum Viride.

7 P. M.—Patient still improving; pulse 73, soft and full; skin warm and moist, perspiration.

Treatment the same.

11th. 7 A. M. Patient not so well; pulse 75, hard and chorded; skin dry and hot; tongue red and dry; mind delirious; more stupor; bowels open: on enquiry, found, through misunderstanding, the nurse had omitted the Veratrum Viride.

Former treatment to be renewed; fresh vesicants applied.

7 P. M.—Patient better; pulse 70, soft and regular; skin warm and moist; mind more composed; had slept during the day. Continue treatment.

12th. 8 A. M.—Patient still improving; pulse 70, soft and regular; mind clear; pupils susceptible to light, which, until this time, had been contracted. Same treatment.

The patient continuing to improve daily; his mind becoming clear and conversant; appetite improved; secretions healthy, until the morning.

16. 7 A. M.—Patient complains of head ache; pulse 90, strong and full; slight wandering; restless bowels—had not acted the night before; skin dry and hot; throbbing temples; tongue dry and red; unable to pass urine; to be relieved by the catheter.

℞—Oleum Ricini—to be followed by injection—Veratrum Viride, viii. M. every three hours; cups to temples; counter irritants.

5 P. M.—Called in a hurry to see patient; found him convulsed, breathing at intervals, and difficult; skin dry and hot; pupils contracted; tongue dry; bowels had not acted.

℞—Oleum Tiglii, ii. M. placed on tongue; counter irritants; cups to temples, and all without relief to the patient, he passing into profound coma, perfectly unsusceptible to the action of medicine, gradually declining until the morning of the

19th. 5 A. M.—Patient died convulsed.

[We would suggest to our friends, that their reports of fatal cases would be much more satisfactory, if they would embrace, in details, the *post mortem developments.*—EDS.]

